# Human Fetal Liver Stromal Cells That Overexpress bFGF Support Growth and Maintenance of Human Embryonic Stem Cells

**DOI:** 10.1371/journal.pone.0014457

**Published:** 2010-12-30

**Authors:** Jiafei Xi, Yunfang Wang, Peng Zhang, Lijuan He, Xue Nan, Wen Yue, Xuetao Pei

**Affiliations:** Stem Cell and Regenerative Medicine Lab, Beijing Institute of Transfusion Medicine, Beijing, China; Universidade Federal do Rio de Janeiro, Brazil

## Abstract

In guiding hES cell technology toward the clinic, one key issue to be addressed is to culture and maintain hES cells much more safely and economically in large scale. In order to avoid using mouse embryonic fibroblasts (MEFs) we isolated human fetal liver stromal cells (hFLSCs) from 14 weeks human fetal liver as new human feeder cells. hFLSCs feeders could maintain hES cells for 15 passages (about 100 days). Basic fibroblast growth factor (bFGF) is known to play an important role in promoting self-renewal of human embryonic stem (hES) cells. So, we established transgenic hFLSCs that stably express bFGF by lentiviral vectors. These transgenic human feeder cells — bFGF-hFLSCs maintained the properties of H9 hES cells without supplementing with any exogenous growth factors. H9 hES cells culturing under these conditions maintained all hES cell features after prolonged culture, including the developmental potential to differentiate into representative tissues of all three embryonic germ layers, unlimited and undifferentiated proliferative ability, and maintenance of normal karyotype. Our results demonstrated that bFGF-hFLSCs feeder cells were central to establishing the signaling network among bFGF, insulin-like growth factor 2 (IGF-2), and transforming growth factor β (TGF-β), thereby providing the framework in which hES cells were instructed to self-renew or to differentiate. We also found that the conditioned medium of bFGF-hFLSCs could maintain the H9 hES cells under feeder-free conditions without supplementing with bFGF. Taken together, bFGF-hFLSCs had great potential as feeders for maintaining pluripotent hES cell lines more safely and economically.

## Introduction

Human embryonic stem (hES) cells are pluripotent cells derived from the inner cell mass of blastocyst-stage human embryos [1], [2]. They can be expanded indefinitely as undifferentiated cells for extended periods of time, and possess the capacity to generate all cell types in the body. To date, many of the studies have highlighted the success in differentiating cell populations from hES cells for cell replacement therapy [3], [4]. However, in guiding hES cell technology toward the clinic, one key issue to be addressed is to culture and maintain hES cells much more safely and economically in large scale. hES cells are most commonly maintained on inactivated mouse embryonic fibroblasts (MEFs) feeders in medium supplemented with knockout serum-replacement (KSR) together with basic fibroblast growth factors (bFGF, or FGF 2). These xeno-support systems run the risk of cross-transfer of animal pathogens from the animal feeder. One example is hES cells cultured with animal products that express Neu5Gc, as a nonhuman sialic acid that triggers an immunogenic response [5].

In order to avoid the use of MEFs, human feeders has been used as an alternative method to maintain hES cells, including embryonic fibroblasts, adult fallopian tube epithelium [6], bone marrow stromal cells [7], foreskin fibroblasts [8], human cell lines (D551/CCL-10, CCL-2552), adult skin cells [9], and placenta cells [10]. But recently, Rajala et al. test nine previously reported xeno-free culture media formats, and conclud that none could maintain the undifferentiated growth of hES cells [11]. So more effective human feeder cells should be selected by comparing each type of feeder cell for their capability to support the growth and maintenance of hES cells. hES cells established on the most effective human feeder cells will apparently promote the development of cell-based therapies.

The other systems to avoid using MEFs as feeders is to use a feeder-free environment that cultures hES cells in special media supplemented with Matrigel matrix [12]or fibronectin [13]. However, some require culture on Matrigel but this contains a variety of extracellular matrix (ECM) components, most likely associated with an ill-defined mixture of growth factors [13], [14], [15]. Recently, successful attempts have been made to develop chemically defined culture medium [16], [17], [18], [19]. In the most present study, the authors introduce a defined serum free medium, hESF9, in which bFGF was the only protein components [19]. Nevertheless, there is no consensus as to the optimal formulation of the chemically defined medium. Moreover, it is likely that feeder-free culture are not optimal for developing transplantable hES cell derivative, for feeder-free cultures usually display a higher degree of spontaneous differentiation than conventional culture. And feeder-free systems, using bFGF and other additional growth factors, will significantly increase the cost of the hES cell culture. So, it is not suitable to use in large scale expansion of hES cells for clinical applications. For the time being, the use of feeder cells is still the safest and most cost-effective option to derive and propagate stable hES cells.

Despite the evolution of these culture conditions and the addition of several factors, all require supplementation with bFGF to sustain hES cell potential. bFGF appears to be of similar importance for hES cells self-renewal as leukemia inhibitory factor (LIF) is for mouse ES cells [20]. But the basis to using bFGF to maintain hES cells still remains unknown. In a previous study, very high concentrations of bFGF (up to 100 ng/ml) was used to maintain hES cells [21], this suggests that either bFGF is operating through an receptor that it is relatively unstable or inefficiently presented to the cells in the culture conditions used. On the other hand, it is supposed that bFGF acts on MEFs to release supportive factors and to reduce differentiation- inducing activity. It is suggested that that bFGF, at least in part, promotes self-renewal of hES cells by modulating the expression of transforming growth factor β (TGF-β) ligands, which, in turn, act on hES cells in autocrine manner [22]. In a most present study, insulin-like growth factor 2 (IGF-2) is expressed by autologously derived hES cell fibroblast-like cells (hdFs) in response to bFGF, and alone was sufficient in maintaining hES cell cultures. This study establishes that hdFs produced by hES cells themselves define the stem cell niche of pluripotent human stem cells. The study reveals a previously unappreciated but essential celluar interplay that establishes a paracrine signaling as being required for pluripotency of hES cells [23]. So, it would be possible to establish more effective hES cell culture system by mimicking the stem cell niche for pluripotency.

In the present study, we had now investigated the culture of H9 hES cells on a new human feeder cells—human fetal liver stromal cells (hFLSCs). The new feeder cells permitted H9 hES cells prolonged culture in an undifferentiated state. In particular, we had established a transgenic feeder cells—bFGF-hFLSCs that stably express bFGF by lentiviral systems, which could be used to maintain H9 hES cells without any exogenous growth factors. And the bFGF-hFLSCs specifically express high levels of bFGF and IGF-2, which are the key factor supports hES cells in culture. And hES cells are successfully maintained feeder-free with conditioned medium of bFGF-hFLSCs (bFGF-hFLSCs-CM). Thus, bFGF-hFLSCs were a novel population that was capable of supporting hES cells expansion more effectively and economically. And also the new culture system could be used as an in vitro model to study comprehensive mechanisms of hES cell self-renewal and pluripotency.

## Materials and Methods

### Isolation of hFLSCs

hFLSCs were obtained from 14 weeks human fetal liver tissues as described previously [24]. Human tissue collection for research purpose was approved by Research Ethics Committee of Beijing Institute of Transfusion Medicine. Pregnant women gave written consent for clinical procedure and research use of the embryonic tissues in accordance with the Declaration of Helsinki. hFLSCs were cultured in a medium comprising 45% Dulbecco's modified Eagle's medium (DMEM, Sigma-Aldrich, St. Louis), 45% DMEM/F12 (Sigma-Aldrich), and supplemented with 10% FCS (fetal calf serum; Gibco, Grand Island, NY). Briefly, hFLSCs were prepared using a ceiling culture method. Human fetal liver tissues were minced into 1 mm^3^ pieces, and transferred onto human gelatin-coated 25-cm^2^ flasks. 4 mL of hFLSCs culture medium was added onto the ceiling surface, and after 5 h the flasks were turned back over. The tissue pieces were incubated undisturbed for 7 days at 37°C, allowing the hFLSCs to migrate and adhere to the gelatin-coated surface. Once the primary hFLSCs reached confluency, the hFLSCs were digested with 0.25% Trypsin (Sigma-Aldrich) for 5 min at 37°C. After adding hFLSCs medium, cultures were pipetted into single-cell suspensions, centrifuged, resuspended, and replated onto new flasks.

### Establishment and Characterization of Transgenic hFLSCs that Overexpressing bFGF

hFLSCs that stably express bFGF were established as previously described [24]. Briefly, bFGF cDNA was cloned from human Mesenchymal stem cells (MSCs) using RT-PCR. Then the amplified human bFGF fragments was ligated into a pBPLV lentiviral vector plasmids containing eGFP [25] (kindly provided by Dr. Luigi Naldiai, Vita Salute San Raffaele University, Italy) (supporting information Fig. S1). The lentiviruses were assembled in 293-FT cells lines (Invitrogen). hFLSCs were seeded at 5×10^5^ cells per 25-cm^2^ flasks one day before transduction. The medium was replaced with virus-containing supernatant supplemented with 4 µg/ml polybrene (Sigma-Aldrich), and incubated for 48 h. After 48 h of transfecction, the eGFP positive hFLSCs that constitutively expressed bFGF were sorted by fluorescence-activated cell sorting (FACS) according to the low and high intensity of eGFP expression.

### General hES Cell Culture

hES cells (H9; WiCell Research Institute, Madison, WI) were cultured according to the supplier's instructions. Briefly, hES cells were routinely cultured on CD1 MEFs in knockout (KO)- DMEM/F12 (Invitrogen, Carlsbad, CA) containing 20% KSR(Invitrogen), 2 mM L-glutamine, 100 µM nonessential amino acids (NEAA, Invitrogen), 100 µM β-mercaptoethanol (Invitrogen), 4 ng/mL bFGF (Peprotech, Rocky Hill, NJ) and 0.5% penicillin and streptomycin. The medium was changed every day.

### Culture of hES Cells on hFLSCs and bFGF-hFLSCs

The hFLSCs with or without the bFGF transgene were irradiated (40 Gy of ^60^Co-γ) within twenty generations and frozen in liquid nitrogen. Before passaging, the hFLSCs were thawed and replated into 6 well cell culture plate at the density of 1×10^6^ per well to be feeders. After 24 h, the culture medium was replaced with hES cell culture medium and hES cells could be seeded on the feeders. hES cells cultured on hFLSCs or bFGF-hFLSCs were passaged every one week by mechanical harvesting.

hES cell culture medium for hFLSCs feeders was identical to the medium used in MEFs feeders, and the medium were changed daily. hES cell culture medium for bFGF-hFLSCs feeders was also identical to the medium used in MEFs feeders but removal of the bFGF, and the medium were changed daily by partial medium changes.

### Characterization of hES cells by Flow Cytometry

Single-cell suspensions were made from undifferentiated hES cells. Stained cells were analysed on a FACS Calibur using Cell Quest software (BD Biosciences, San Diego). Unconjugated antibody against Stage-specific embryonic antigen 4 (SSEA4) (Chemicon, Temecula, CA), tumor-related antigen 60 (TRA-1-60) (Chemicon), and OCT4 (Chemicon) were used to viably identify hES cells. Then hES cells were washed twice prior to staining with fluorescein isothiocyanate (FITC)-conjugated goat anti-mouse IgG (BD Biosciences) for 30 min. Finally, the cells were washed twice with PBS and analyzed by flow cytometery. Negative controls represented staining of cells in the absence of primary antibody.

### Embryoid Bodies (EBs)-mediated In Vitro Differentiation of hES Cells Cultured on bFGF-hFLSCs

For EBs formation, H9 hES cells were harvested by treating with collagenase IV. The clumps of the cells were transferred to 6-well Ultra-Low Attachment Plate (Corning, NY) in DMEM/F12 containing 20% KSR (Invitrogen), 2 mM L-glutamine, 100 µM NEAA (Invitrogen), 100 µM-mercaptoethanol (Invitrogen), and 0.5% penicillin and streptomycin. The medium was changed every other day. After 6 days as a floating culture, EBs were transferred to gelatin-coated plate and cultured in the same medium for another 10 days.

### Teratoma formation

Approximately 5×10^6^ H9 hES cells cultured on bFGF-hFLSCs for 10 passages (about 70 days) with undifferentiated morphology were subcutaneously injected into the rear leg armpit of 6–8 weeks old Nonobese Diabetic/Severe Combined Immunodeficiency (NOD-SCID) mice. About 6–10 weeks after injection, teratoma formed and were dissected and fixed with 4% paraformaldehyde overnight. The samples were then cut at a thickness of 10 µm and transferred to Poly-D-Lysine-coated slides. Sections were examined histologically after hematoxylin and eosin staining. All the animal experiments were approved by the Animal Care and Use Committee of Beijing Institute of Transfusion Medicine.

### Immunocytochemistry

Cells were rinsed twice with PBS before fixation with 4% paraformaldehyde, followed by protein staining before permeabilization with 0.2% Triton X-100 or 100% methanol and staining for OCT4. Cells were blocked with 10% normal rabbit serum or 10% normal goat serum at room temperature. Antibodies used were anti-IGF1R (R&D Systems, Minneapolis), anti-FGFR1 (Chemicon), anti-OCT4 (Chemicon), anti-SSEA-4 (Chemicon), anti-TRA-1-60 (Chemicon), anti-TRA-1-81 (Chemicon), anti-βIII-TUBULIN (BD Biosciences), anti-α-SMA (BD Biosciences), and anti-AFP (Sigma-Aldrich). Cells were incubated with primary antibodies followed by secondary detection with FITC-conjugated goat anti-mouse IgG (BD Biosciences), or FITC-conjugated rabbit anti-goat IgG (BD Biosciences), or TRITC-conjugated goat anti-mouse IgG (BD Biosciences). Nucleuses were stained with 1 µg/ml DAPI (Sigma-Aldrich). Fluorescence was visualized using Olympus IX70 (Olympus, Tokyo) fluorescence microscope, and images were recorded using a digital SPOT RT3 CCD camera (Diagnostic Instruments inc., Sterling Heights, MI) using version 4.5 SPOT software.

### Feeder-free maintenance of hES cells in bFGF-hFLSCs-CM)

hES cells lines H9 were maintained in a feeder-free culture over matrigel-coated (BD Biosciences) six-well plate in bFGF-hFLSCs-CM. bFGF-hFLSCs-CM was prepared and collected as described in detail for MEF-CM [12], [26]. The basal media used to prepare the CM consisted of 80% KO-DMEM/F12 supplemented with 20% KSR, 1% NEAA, 1 mM L-glutamine, 0.1 mM β-mercaptoethanol. H9 hES cells was maintained feeder-free for 10–15 passages (about 70–100 days). The media was changed daily and the hES cells cultures were split (1:3) using collagenase IV. H9 hES cells were also cultured under feeder free conditions using MEF-CM [26] and mTeSR1(StemCell Technologies, Vancouver, BC, Canada) following the manufacturer's instructions.

### Reverse Transcription-Polymerase Chain Reaction Analysis

Total RNA was extracted by TRIzol reagent (Invitrogen) according to the manufacturer's instructions. 1 µg RNA was then reverse-transcribed into cDNA by Avian Myeloblastosis Virus (AMV) reverse transcriptase (Takara Bio, Shiga, Japan). PCR was performed with rTaq polymerase (TaKaRa). An aliquot of PCR products was analyzed on 1.5% ethidium bromide-stained agarose. Glyceraldehyde-3-phosphate dehydrogenase (GAPDH) or β-actin was used as a housekeeping gene to evaluate and compare quality of different cDNA samples.

### RT-PCR Primer Sequences

The following primer pairs were used for the amplification of target mRNAs: Oct-4 (123 base pair [bp]), forward 5′- AAC CTG GAG TTT GTG CCA GGG TTT-3′ and reverse 5′- TGA ACT TCA CCT TCC CTC CAA CCA-3′; Sox-2 (151 bp), forward 5′- GGG AAA TGG GAG GGG TGC AAA AGA GG-3′ and reverse 5′- TTG CGT GAG TGT GGA TGG GAT TGG TG-3′; Nanog (216 bp), forward 5′- CAG AAG GCC TCA GCA CCT-3′ and reverse 5′- CTG TTC CAG GCC TGA TTG TT-3′; HCG (510 bp), forward 5′- CAG GGG ACG CAC CAA GGA TG-3′ and reverse 5′- GTG GGA GGA TCG GGG TGT CC-3′; CK18 (233 bp), forward 5′- AGC TCA ACG GGA TCC TGC TGC ACC TTG-3′ and reverse 5′- CAC TAT CCG GCG GGT GGT GGT CTT TTG-3′; AFP (281 bp), forward 5′- GAA TGC TGC AAA CTG ACC ACG CTG GAA C-3′ and reverse 5′- TGG CAT TCA AGA GGG TTT TCA GTC TGG A-3′; Brachyury (144 bp), forward 5′- TGT CCC AGG TGG CTT ACA GAT GAA-3′ and reverse 5′- GGT GTG CCA AAG TTG CCA ATA CAC-3′; PAX6 (317 bp), forward 5′- ACC CAT TAT CCA GAT GTG TTT GCC CGA G-3′ and reverse 5′- ATG GTG AAG CTG GGC ATA GGC GGC AG-3′; GFAP (328 bp), forward 5′- GGC CCG CCA CTT GCA GGA GTA CCA GG-3′ and reverse 5′- CTT CTG CTC GGG CCC CTC ATG AGA CG-3′; bFGF (486 bp), forward 5′- AAC TGC AGA TGC CAG CCG GGA GCA TC-3′ and reverse 5′- ACG CGT CGA CTC AGC TCT TAG CAG ACA TTG GAA GAA-3′; IGF-1 (184 bp), forward 5′- GCT GGT GGA TGC TCT TCA GTT C-3′ and reverse 5′- AGC TGA CTT GGC AGG CTT GAG-3′; IGF-2 (150 bp), forward 5′- TTG CTC TAC CCA CCC AAG AC-3′ and reverse 5′- GAT GGA ACC TGA TGG AAA CG-3′; TGF-β1 (221 bp), forward 5′- GCG TGC TAA TGG TGG AAA C-3′ and reverse 5′- GCT GAG GTA TCG CCA GGA AT-3′; IGF1 receptor (99 bp), forward 5′- CCA AAG ACA AAA TCC CCA TC-3′ and reverse 5′- CTT TCT CCC CAC CAC ACA C-3′; FGF receptor 1 (119 bp), forward 5′- GGC TAC AAG GTC CGT TAT GC-3′ and reverse 5′- TGG TAT GTG TGG TTG ATG CTG-3′; β-actin (222 bp), forward 5′- GAT CCA CAT CTG CTG GAA GG-3′ and reverse 5′- AAG TGT GAC GTT GACATC CG-3′; GAPDH (325 bp), forward 5′- TGT CAT CAA TGG AAA TCC CAT CAC C-3′ and reverse 5′- CAT GAG TCC TTC CAC GAT ACC AAA-3′.

### Protein Extraction and Western Blotting

Protein extracts were obtained using RIPA lysis buffer (50 mM Tris-HCl pH 7.4; 150 mM NaCl; 1% NP-40; 0.1% SDS; 0.5% Na-deoxycholale) with protease inhibitor (Merk Chemicals, Darmstadt, Germany). Cell lysates were cleared at 12,000 g for 10 minutes at 4°C. Equal amounts of proteins in the supernatants were separated on 12% SDS-PAGE and blotted on PVDF membrane (Bio-Rad, Hercules, CA). Membranes were blocked with 4% non-fat milk (Bio-rad)/TBS-T and incubated with an anti-bFGF antibody or anti-IGF-2 antibodies (all from R&D Systems) followed by horseradish peroxidase (HRP)-conjugated secondary antibodies and visualized with ECL (Santa Cruz Biotechnology, Santa Cruz, CA) detection. Chemoluminescence was visualized on an x-ray film.

### Enzyme-linked Immunosorbent Assays

Free human bFGF was determined with the FGF basic ELISA kit (R&D Systems). Assays were performed according to the manufacturer's recommended protocol. Protein extracted from culture supernatants were standardized for total protein content using the BCA Protein Assay kit (Pierce, Rockford, IL) before analysis. Results are representative of four independent experiments.

### Karyotype Analysis

Karyotype analyses of H9 hES cells cultured on hFLSCs and bFGF-hFLSCs were carried out at passages 15 (about 100 days) using the standard G-banding procedure. The karyotype analyses were done at the Beijing Institute of Radiation Medicine, Cytogenetics Laboratory (Beijing, China).

## Results

### hFLSCs Used as Feeder Cells for Maintenance of H9 hES Cells

**Figure 1:**
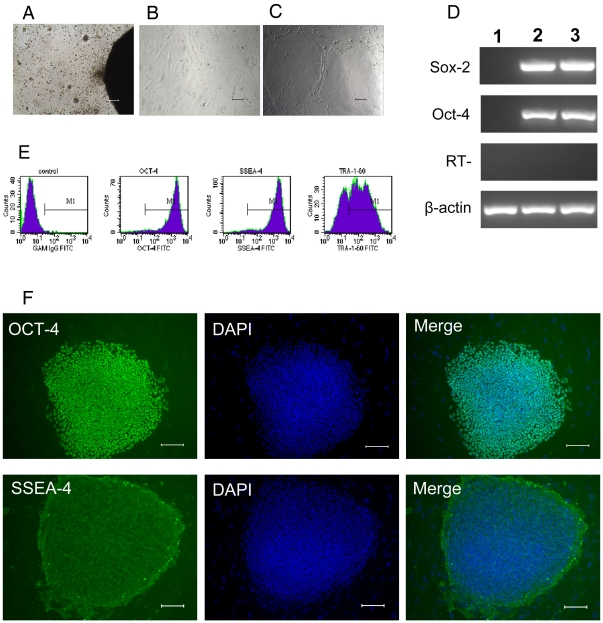
Morphology and characterization of H9 hES cells grown on hFLSCs. (A): Derivation of hFLSCs by ceiling culture method. (B): hFLSCs show typical fibroblast morphology. (C): Morphology of H9 hES cells grown on hFLSCs. (D): RT-PCR analysis of H9 hES cells grown on hFLSCs for 10 passages (about 70 days). 1: hFLSCs feeder cells, 2: H9 hES cells cultured on MEF, H9 hES Cells cutred on hFLSCs. (E): Cell phenotype analysis of H9 hES cells that cultured on hFLSCs for 10 passages (about 70 days) by flow cytometry.(F): H9 hES cells grown on hFLSCs for 15 passages (about 100 days) stained with antibody recognizing the OCT-4 and SSEA-4. Nuclei were stained with DAPI (blue). Bars: (A-C, F) 100 µm.

We isolated and cultured hFLSCs from 14 weeks human fetal liver. About one week in culture the stromal cells migrated from the border of the tissue clumps (Fig. 1A), and it took two weeks for the cells to become confluent. After the primary and three subsequent passages, hFLSCs were adherent to be fusiform shape and highly uniform in morphology (Fig. 1B). As hFLSCs are propagated in culture for more than 30 passages (about 90 days), there are no obviously cell morphology changes. And then the irradiated hFLSCs were used for feeder cells to maintain H9 hES cells. Thus far, H9 cells were grown on hFLSCs for over 15 passages (about 100 days). We found that H9 hES cells colonies grown on hFLSCs were dense and compact, and they exhibited the typical morphology of hES cells (Fig. 1C). RT-PCR analysis showed positive expression of OCT-4, SOX-2 (Fig. 1D) when growing on hFLSCs for 10 passages (about 70 days). Flow cytometry analysis revealed that H9 hES cells grown on hFLSCs expressed hES specific markers OCT-4, SSEA-4, and TRA-1-60 (Fig. 1E). Immunocytochemical analysis also showed that H9 hES cells cultured on hFLSCs for 15 passages (about 100 days) expressed markers typical of hES cells, such as OCT-4 and SSEA-4 (Fig. 1F). The H9 hESCs cultured on hFLSCs had no differences with cultured on MEF (supporting information Fig. S2A–C) Karyotyping of the hES cells showed that H9 hES cells grown on hFLSCs had normal female karyotype (supporting information Fig. S3).

### Maintenance of H9 hES cells on bFGF-hFLSCs Feeder Cells

We have established the bFGF-hFLSCs by lentiviral system in our previous study [24]. After having been transfected with bFGF, the hFLSCs were sorted by FACS according to the intensity of eGFP expression (supporting information Fig. S4). The transduced hFLSCs with high eGFP expression were named as bFGF-hFLSCs and used as new feeder cells for maintenance of hES cells. As bFGF-hFLSCs were propagated in culture, there were also no obviously cell morphology changes. Flow cytometry analysis was used to characterize cell surface markers of bFGF-hFLSCs, showing that the bFGF-hFLSCs were positive for some stromal progenitor markers, such as CD29, CD44, CD71, CD90, and CD105, but negative for CD34 and CD45 (supporting information Fig. S5), which was similar to the untransfected hFLSCs [24]. bFGF-hFLSCs maintained normal male karyotypes after 20 passages (about 60 days) (supporting information Fig. S6).

**Figure 2:**
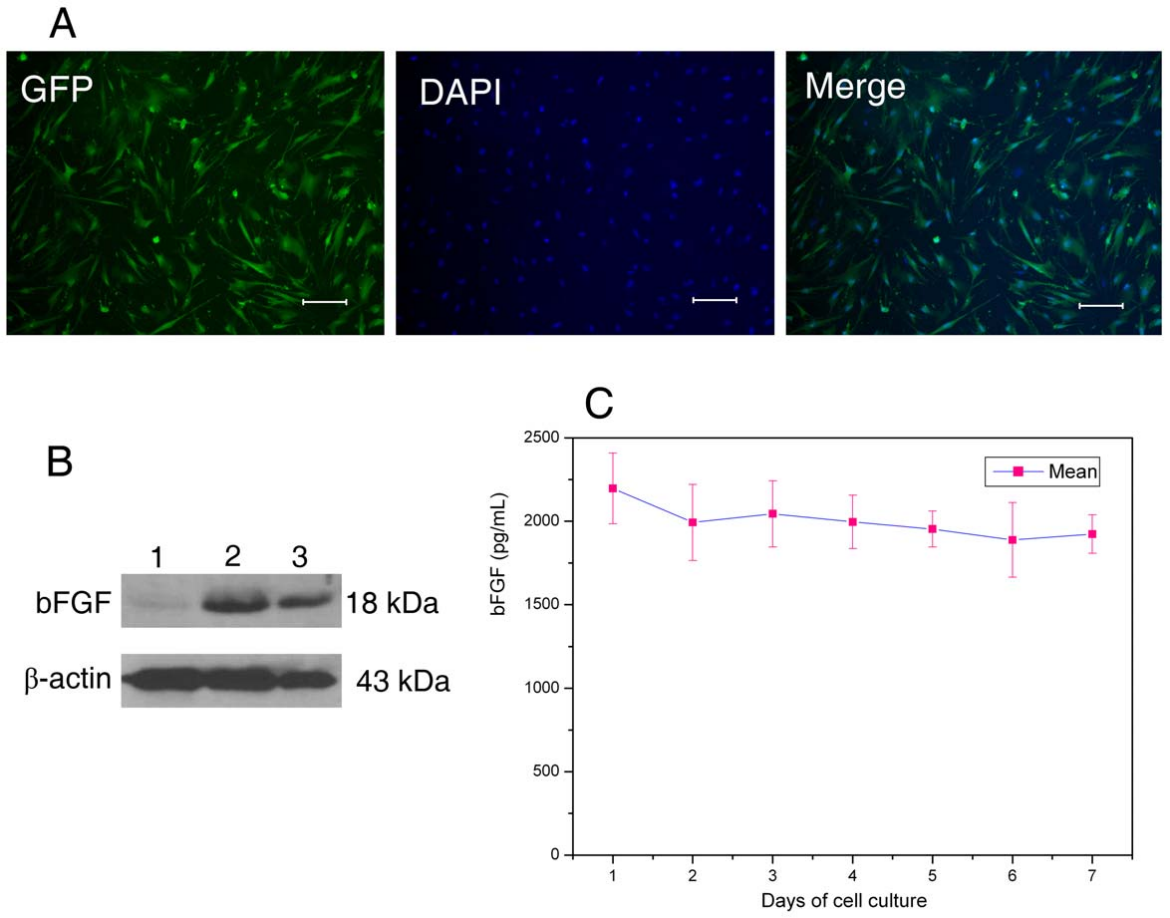
Morphology and identification of bFGF over-expressing hFLSCs feeder cells. (A): eGFP expression of γ-irradiated bFGF-hFLSCs imaged by fluorescence microscope. Bars: 100 µm. (B): Western blotting analysis indicated that bFGF highly expressed in γ-irradiated bFGF-hFLSCs. (lane 1) γ-irradiated hFLSCs transfected with control vectors; (lane 2) γ-irradiated bFGF- hFLSCs; (lane 3) A half amount of the lane 2. (C): The enzyme-linked immunosorbent assay (ELISA) to bFGF showed that bFGF-hFLSCs could stably secret bFGF for one week. Values are presented as means ± SD, n = 4.

The irradiated bFGF-hFLSCs feeder cells showed the typical mesenchymal cell shape, and almost all cells expressed the eGFP (Fig. 2A). To determine the expression of bFGF in bFGF-hFLSCs, western blotting was used to detect the expression level of bFGF protein in bFGF-hFLSCs feeders. As shown in Fig. 2B, there was high bFGF expression in bFGF-hFLSCs feeders. To measure bFGF in cell culture supernates, the quantikine bFGF immunoassay kit was used to determine values accurately. The results showed that soluble bFGF in culture medium of γ-irradiated bFGF-hFLSCs maintained in high level in about one week (Fig. 2C). The secreting bFGF from bFGF-hFLSCs feeder cells might replace the exogenous bFGF in hES cell culture.

**Figure 3:**
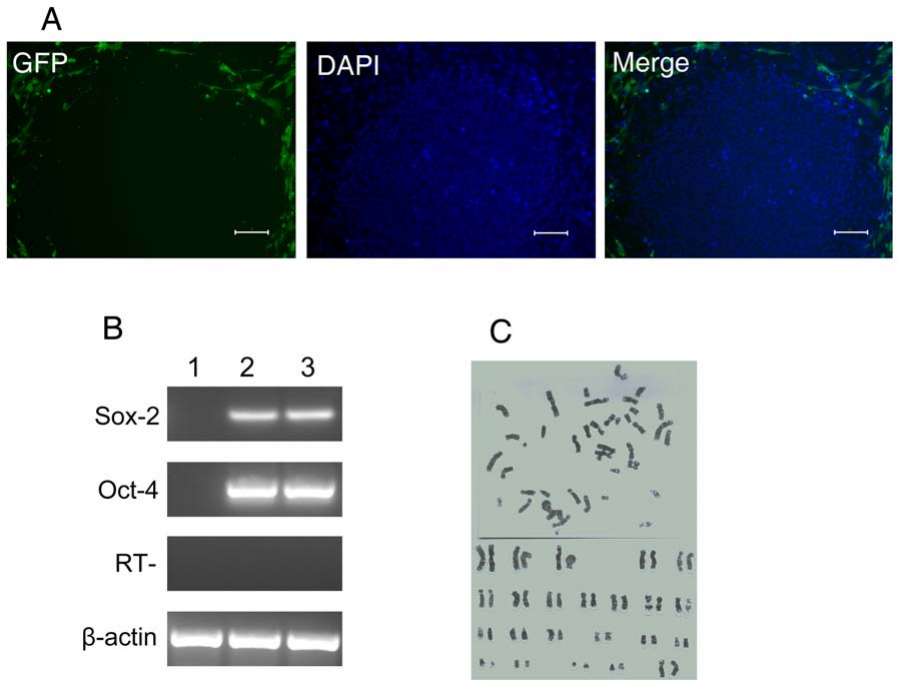
Characterization and karyotyping of H9 hES cells cultured on bFGF-hFLSCs feeder cells. (A): The H9 hES cells cultured on bFGF-hFLSCs imaged by fluorescence microscope. Bars: 100 µm. (B): RT-PCR analysis of H9 hES cells grown on bFGF-hFLSCs for 10 passages (about 70 days). 1: bFGF-hFLSCs feeder cells, 2: H9 hES cells cultured on MEF, H9 hES Cells cutred on bFGF-hFLSCs. (C): H9 hES cells grown on bFGF-hFLSCs for 15 passages (about 100 days) showed normal female karyotype (46, XX).

To test whether bFGF-hFLSCs support undifferentiated growth of hES cells without supplementing with exogenous bFGF, we cultured H9 hES cells on bFGF-hFLSCs. Indeed, most H9 hES cells cultured with bFGF-expressing hFLSCs were maintained in an undifferentiated state. Undifferentiated hES colonies also could be found under fluorescence conditions for eGFP detection (Fig. 3A).

**Figure 4:**
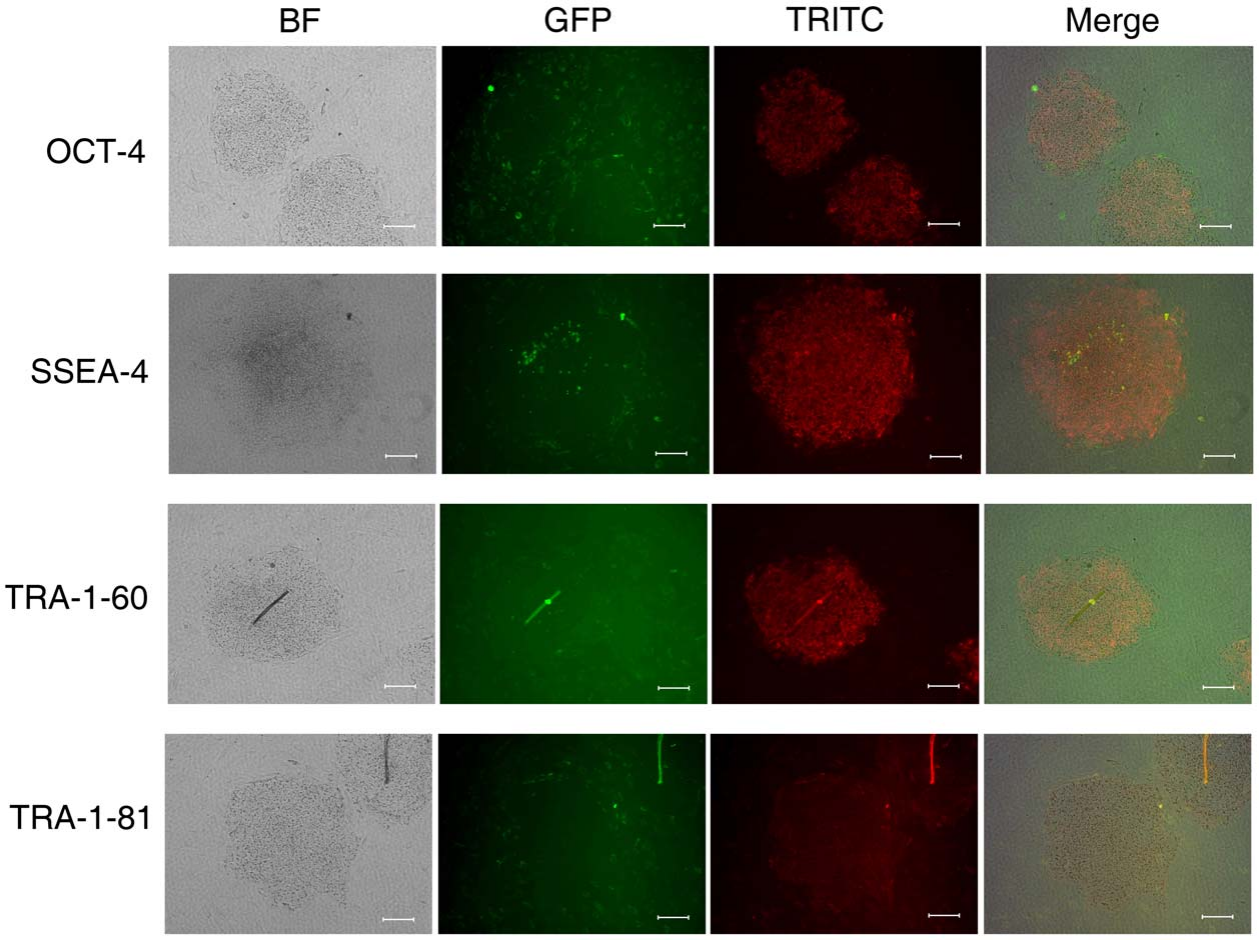
Immunocytochemistry for OCT-4, SSEA-4, TRA-1-60, TRA-1-81 of the H9 hES cells that cultured on bFGF-hFLSCs for 12 passages (about 80 days). Bars: 100 µm.

RT-PCR analysis showed positive expression of OCT-4, SOX-2 (Fig. 3B) when H9 hES cells grown on bFGF-hFLSCs for 10 passages (about 70 days). Karyotype analysis showed that H9 hES cells grown on bFGF-hFLSCs had normal female karyotypes (Fig. 3C). The new culture system proved of supporting the culture of H9 hES cells and the expression of the specific markers OCT-4, SSEA-4, TRA-1-60 and TRA-1-81 was similar to cells grown on MEFs (Fig. 4). Currently, hES cells are commonly cultured on MEF feeders with 4 ng/ml exogenous bFGF in culture conditions. Our data suggested that without the exogenous bFGF, the fetal liver stromal cells with the bFGF transgene provided an environment to maintain hES cells for a long culture time.

### Embryoid Body-Mediated Differentiation of hES Cells Cultured on bFGF-hFLSCs

**Figure 5:**
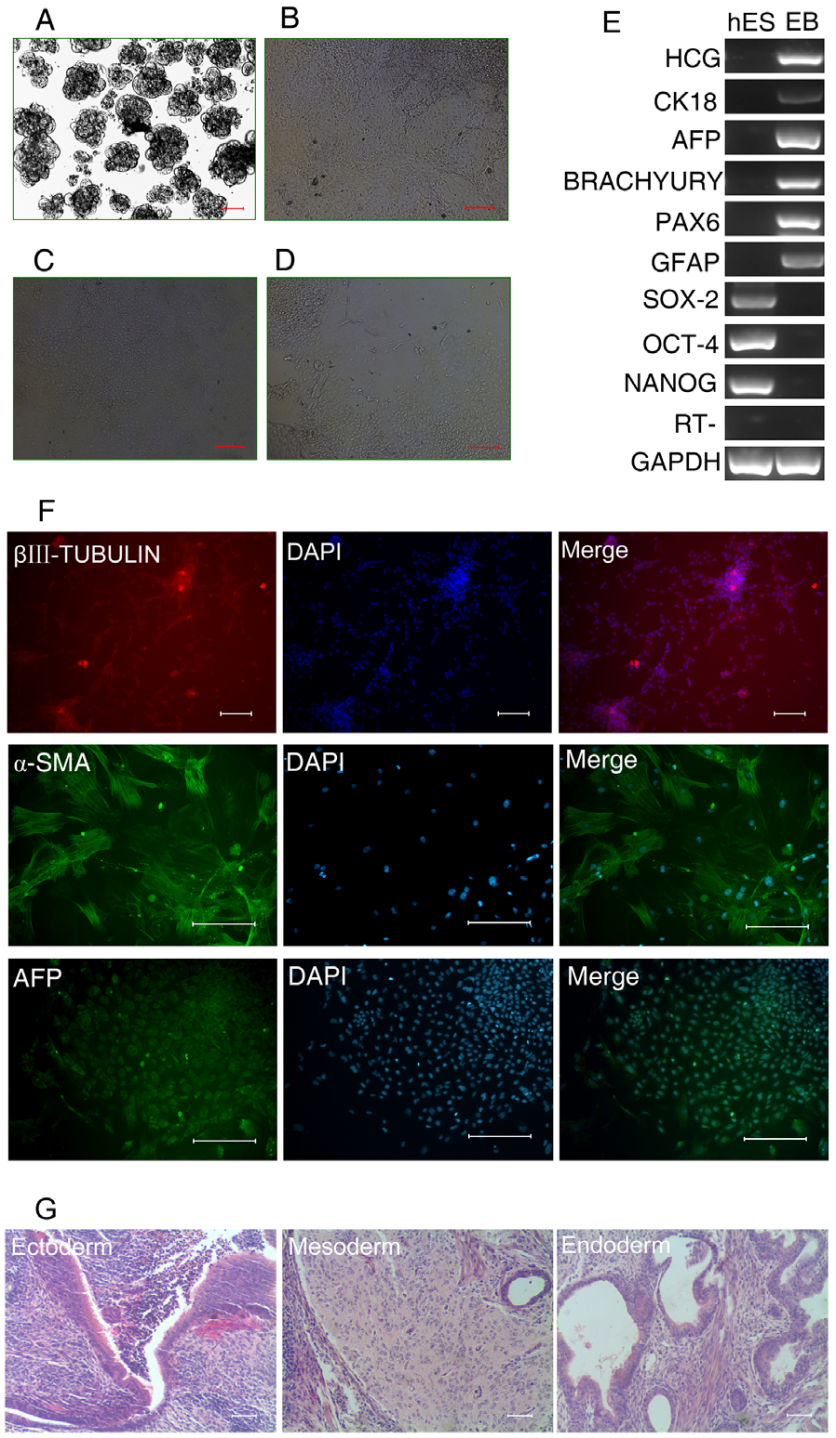
In vitro and in vivo differentiation of H9 hES cells cultured on bFGF-hFLSCs for 10 passages (about 70 days). (A): Floating culture of H9 hES cells at day 6. Bar: 100 µm. (B–D): Images of differentiated cells at day 10 (B), epithelial cells (C), fibroblast like cells (D). Bars: 100 µm. (E): RT-PCR analysis of various differentiation markers for the three germ layers and extraembryonic trophoblast. (F): Immunocytochemistry of AFP, a-SMA, and βIII-TUBULIN. Nuclei were stained with DAPI (blue). Bars: 100 µm. (G): Teratoma formation. bFGF-hFLSCs-cultured H9 cells for 10 passages (about 70 days) form teratoma generating structures representative of three germ layers. Bars: 100 µm.

The hES cells retained the capacity for extensive differentiation as indicated by analysis of ectoderm, mesoderm, and endoderm marker gene expression following EB formation. To determine the differentiation ability of H9 hES cells cultured on bFGF-hFLSCs in vitro, we used floating cultivation to form EBs [27]. After 6 days in suspension culture, H9 hES cells formed ball-shaped EBs (Fig. 5A). The EBs did not contain the eGFP-positive-hFLSCs feeder cells by examining with fluorescence microscopy. We transferred EBs to gelatin-coated plates and continued cultivation for another 10 days (Fig. 5B). Attached cells showed various types of morphologies, such as those resembling epithelial cells and fibroblast like cells (Fig. 5C, D). RT-PCR confirmed that these differentiated cells expressed human chorionic gonadotrophin (HCG, a marker of extraembryonic trophoblast), α-fetoprotein (AFP, endoderm), cytokeratin 18 (CK 18, endoderm), BRACHYURY (mesoderm), and paired box 6 (PAX6, ectoderm), glial fibrillary acidic protein (GFAP, ectoderm) (Fig. 5E). In contrast, expression of OCT4 and SOX-2 was almost undetectable (Fig. 5E). The in vitro differentiation capability of H9 hES cells was similar to that cultured on MEF (supporting information Fig. S2D). Immunocytochemistry detected cells positive for βIII-tubulin (a marker of ectoderm), α-smooth muscle actin (α-SMA, mesoderm), and AFP (endoderm) (Fig. 5F). These data demonstrated that hES cells could differentiate into three germ layers in vitro. We subsequently tested long-term culture of H9 hES cells to 15 passages (about 100 days), morphologies of undifferentiated hES cell colonies were maintained as those cultured on MEFs feeders. Additionally, H9 hES cells cultured on bFGF-hFLSCs for 10 passages (about 70 days) were able to form teratoma comprising cell lineages of three embryonic germ layers in vivo in NOD-SCID mice (Fig. 5G).

### The FGF and IGF Related Receptors Expression in the hES and hFLSCs Culture

**Figure 6:**
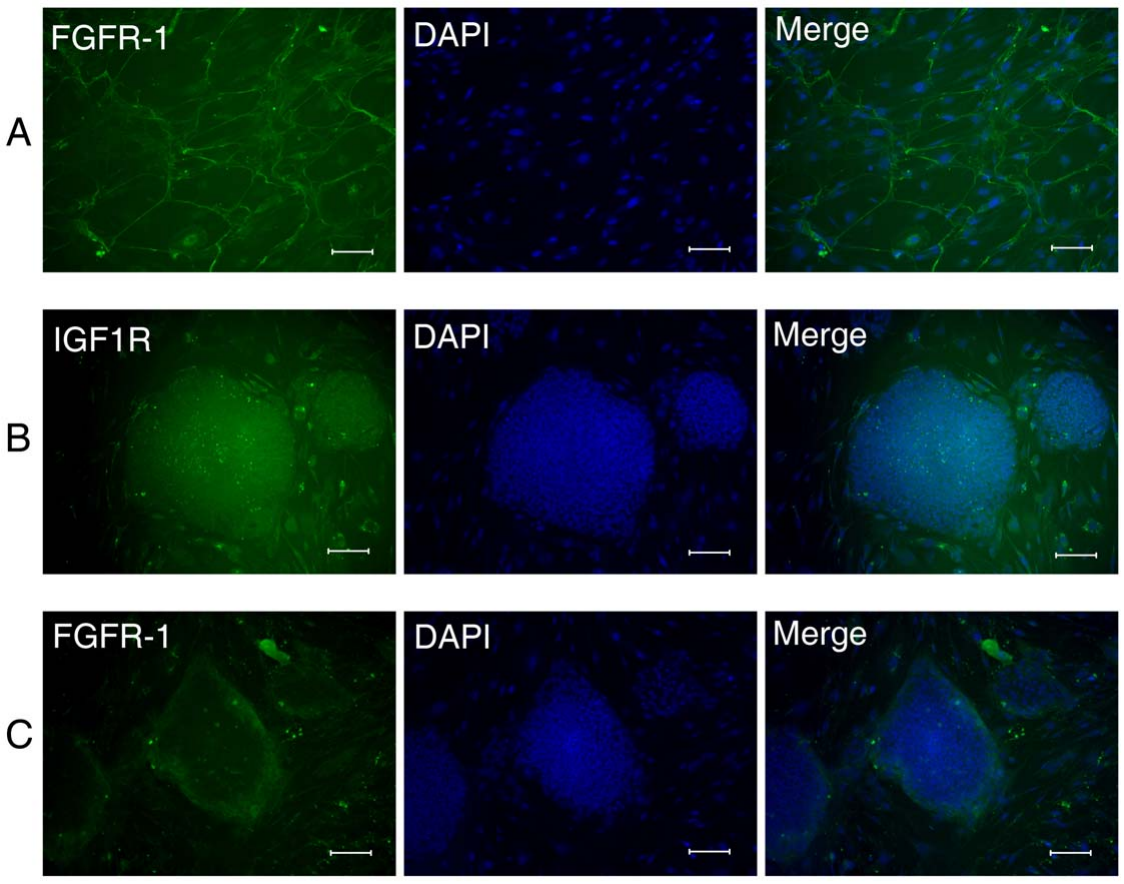
Immunocytochemistry for FGFR1 and IGF1R of the hFLSCs (A) and hES cells that cultured on hFLSCs (B,C). Nuclei were stained with DAPI (blue). Bars: 100 µm.

There are four FGF receptors (FGFR1–4). bFGF is related to FGFR1, which accounts for the high affinity of bFGF binding sites in the nucleus and cytoplasm [28]. Previous study suggest that FGF receptors may be important to hES cell culture systems, but are not dominantly expressed by the self-renewing, pluripotent human stem cells. However, IGF1R expression correlates with pluripotent stem cell markers and thereby underscores both the uniqueness and general importance of the IGF-2/IGF1R axis in hES cell lines [23]. So, in the present study, we investigated expression of FGFR1 and IGF1R to confirm the link between bFGF and IGF in the H9 hES cells and hFLSCs culture. As shown in Fig. 6A, the hFLSCs expressed FGFR1, and didn't express IGF1R. We also observed that IGF1R expressed exclusively within H9 hES cell colonies (Fig. 6B), while FGFR1 expression was limited to cells surrounding the H9 hES cell colonies (Fig. 6C). These results provided another example of bFGF's role in the stem cell niche underlining human feeder cells indirectly regulating hES cells.

### bFGF-hFLSCs Expressed IGF-2 and Other Related Factors

**Figure 7:**
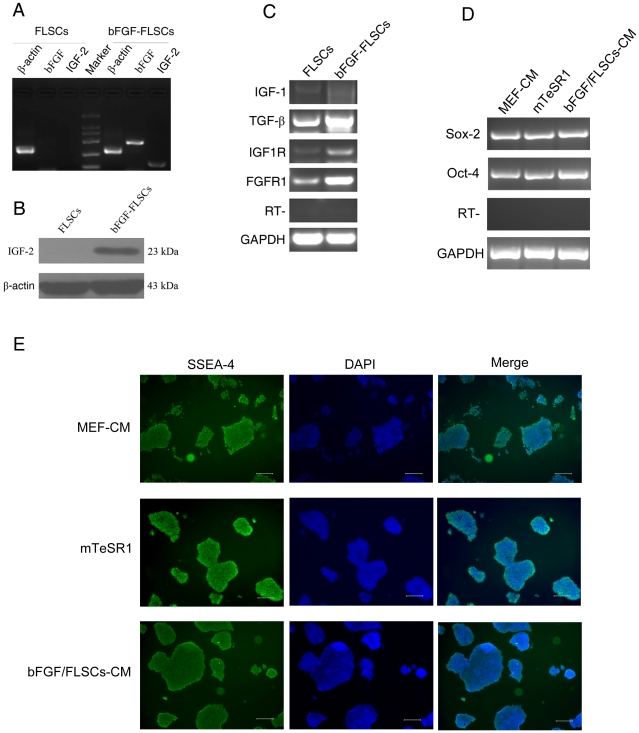
H9 hES cells could be maintained feeder-free with bFGF-hFLSCs-CM. (A): RT-PCR analysis of bFGF and IGF-2 gene expression in hFLSCs and bFGF-hFLSCs. (B): Western blotting analysis of IGF-2 gene expression in hFLSCs and bFGF-hFLSCs. (C): RT-PCR analysis of bFGF and IGF-2 related gene expression in hFLSCs and bFGF-hFLSCs. (D): RT-PCR analysis of H9 hES cells grown on MEF-CM, mTeSR1 and bFGF-hFLSCs-CM for 10 passages (about 70 days). (E): Immunophenotypic characterization of H9 hES cells maintained feeder-free with MEF-CM, mTeSR1 and bFGF-hFLSCs-CM for 10 passages (about 70 days). Nuclei were stained with DAPI (blue). Bars: 250 µm.

Since bFGF is known to regulate IGF-1 [29] and IGF-2 [30], we examined the relative expression level of IGF-2 genes in bFGF-hFLSCs by RT-PCR and western blotting. The results showed that both the expression of bFGF and IGF-2 mRNA highly increased in bFGF-hFLSCs (Fig. 7A). Western blotting also showed that the IGF-2 protein was highly induced in bFGF-hFLSCs (Fig. 7B). These data indicated that bFGF-hFLSCs produced adequate amounts of endogenous IGF-2 to maintain hES cells. To determine whether bFGF influences not only IGF-2, but also other related factors, we investigated the expression of IGF-1, TGF-β1, IGF1R and FGFR1 by RT-PCR in bFGF-hFLSCs. TGF-β1 expression was shown to be induced by bFGF transfection. These data suggested that bFGF-hFLSCs produced TGF-β1 factors required by hES cells to maintain an undifferentiated state. And also FGFR1 and IGF1R expression of bFGF-hFLSCs was also higher than that of hFLSCs transfected with control vector.

### Feeder-free maintenance of hES cells in bFGF-hFLSCs-CM

Many feeder-free hES cells culture systems is still based on the use of xenogenic MEF-CM. Only in very recent work have hES cells been maintained feeder free using chemically defined medium [16], [19]. We therefore sought to determine whether H9 hES cells can be maintained pluripotent and feeder-free in bFGF-hFLSCs-CM. H9 hES cells were maintained for 15 passages (about 100 days) feeder-free in bFGF-hFLSCs-CM. No differences in culture homeostasis were observed among hES cells maintained in bFGF-hFLSCs-CM versus MEF-CM and mTeSR1. Identical to H9 hES cells cultured in MEF-CM and mTeSR1, those maintained in bFGF-hFLSCs-CM retained typical hES cell morphology and expression of transcription factors Oct4 and Sox-2 (Fig. 7D). Similarly, both H9 hES cells expressed the the pluripotency associated surface markers SSEA-4 (Fig. 7E).

## Discussion

Here we described a new population of hFLSCs that were capable of supporting the H9 hES cells expansion in vitro. We had cultured H9 hES cells through multiple passages while they retained stable expression of undifferentiated markers of hES cells, and a capacity to differentiate to three germ layer cells. We further showed that hFLSCs over-expressing bFGF could be used as a new feeder cells to maintain H9 hES cells without supplementing with any exogenous growth factors. And the conditioned medium of bFGF-hFLSCs could maintain the H9 hES cells under a feeder-free conditions without supplementing with bFGF. Our results demonstrated that all undifferentiated H9 hES cells expressed IGF1R hFLSCs express FGFR1. And the bFGF-hFLSCs specifically expressed high levels of bFGF, IGF-2 and other related factors to support hES cells in culture. We suggested that bFGF-hFLSCs maintained H9 hES cells by establishing a stem cell niche through which supporting the self-renewal and pluripotency of hES cells.

Mouse feeder cells are associated with risks such as viral infection and pathogen transmission when hES cells are used in clinical trials. Various culture conditions have been developed to overcome these problems. Previous studies have used certain types of primary human cells, as well as commercially available cell lines, as feeder layers [9], [31], [32], [33]. These studies all show that human feeder cells can be used to support hES cell growth and miantainenane. In our study, we isolated hFLSCs from 14 weeks fetal liver tissues, thus probably providing embryonic-like niche for hES cells. Our results showed that H9 hES cells can be maintained on hFLSCs feeder cells for over 15 passages (about 100 days). hFLSCs could be used as a new human feeder cell to support hES cells expansion in vitro.

One of the improved culture conditions is a feeder-free culture [12], [15]. And several chemically defined culture mediums also are used to maintain hES cells [17], [19], [34]. One important issue is that these culture mediums are so expensive that they can't be used in large-scale expansion of hES cells for prospective clinical applications. It will be very important to find more economical hES cell culture methods.

As we have known, bFGF remains a necessary growth factor for the undifferentiated growth of hES cells. It regulates the expression of TGFβ1, Activin A and the BMP4 antagonist which are important for hES cell growth and self-renewal [22], [35]. The fact that MEF and human feeder cell lines secrete varying amounts of bFGF may partially explain why different batches of MEFs and human feeder cell lines support hES cells differently [35]. Cai and colleagues generate immortalized feeder cells overexpressing Wnt3a that are supportive of hES cell expansion and pluripotency maintenance [36]. One fibroblast-like feeder cell line which differentiated spontaneously from hES cells secreting bFGF supported hES cell expansion particularly well up to passages 15 [37]. In this study about 11.66±0.23 pg/ml of FGF2 is detected in the germ layer derived fibroblast cells (GLDF) conditioned medium. These studies indicated that using gene modification feeder cells might improve the hES cell culture method. Our results showed that bFGF-hFLSCs secreted much higher bFGF than in the previous study [37]. We found that the maintenance of undifferentiated hES cells could be promoted by the bFGF-hFLSCs without supplementing with growth factors. The hES cell colonies maintained on bFGF-hFLSCs showed less signs of morphological differentiation. The hES cells were positive for the pluripotent markers when maintained on bFGF-hFLSCs. hES cells were stably passaged for up to 15 passages (about 100 days) by co-culture with bFGF-hFLSCs.

Expression of bFGF in bFGF-hFLSCs feeder cells made the hES cell culture more economical where supplemental bFGF is not needed. It was quite evident that bFGF-hFLSCs cells presented to hES cell researchers with a novel feeder system for stable hES cell cultures. bFGF-hFLSCs were eased to culture for large scale, and enabled the development of goods mamufacturing practice (GMP) systems. bFGF-hFLSCs or its conditioned media could be an efficient alternative and useful to support the derivation and culture of new hES cell lines for clinical applications as comparable to hES cells cultured on MEFs.

Several recent studies have shown that IGF-2 play an important role in maintenance of hES cells [23], [34], [38]. IGF-2 has been suggested to play important roles in embryonic but not adult growth and development [39], [40]. IGF-2 has also been used to establish pluripotential cell lines [41] and maintain undifferentiated stem cells from the inner cell mass in culture [41], [42]. In a previous study [43], the author identified IGF-2 expressed in a novel fetal liver cell populations as a novel stem cell growth factor. Because IGF-2 has pleiotropic effects, it may have the potential to act on a broader spectrum of stem cells, both ex vivo and in vivo. One study focus on FGF receptor expression profiles in hES cell cultures, and suggest that bFGF may stimulate undifferentiated hES cell proliferation directly or indirectly [44]. In another study, a simultaneous interrogation of 42 receptor tyrosine kinases (RTKs) in hES cells following stimulation with MEF conditioned medium (CM) reveal rapid and prominent tyrosine phosphorylation of insulin receptor (IR) and IGF1R [34]. In the most recent study, the authors showed that hES cells spontaneously and continuously differentiate into hdFs, providing a continuous source of endogenous hES cell supportive factors, including IGF-2 and a host of TGF-β family of factors and other ligands [23]. On the whole, these studies demonstrate a direct role of the IGF-2/IGF1R axis on hES cell self-renewal and pluripotency.

In the present study, we suggested bFGF and IGF-2 as the principal growth factor produced by bFGF-hFLSCs that supports expansion of H9 hES cells. And our results showed that hFLSCs expressed FGFR1, which could be stimulated by the bFGF secreted by bFGF-hFLSCs through autocrine and paracrine model. And after transducing with bFGF, the expression of IGF1R, FGFR1, and TGF-β1 in bFGF-hFLSCs was much higher than in hFLSCs. This positive model made bFGF-hFLSCs express sufficient IGF-2 and TGF-β1 to support growth and maintenance of hES cells through IGF-2/IGF1R axis and other undefined signal pathways. bFGF-hFLSCs feeder cells were central to establishing the signaling network among bFGF, IGF-2, and TGF-β, and thereby probably providing the framework in which hES cells were instructed to self-renew or to differentiate.

The concept that a regulatory niche creates an essential and supportive microenvironment for stem cells has been well established in several organisms and lineages [45]. In our previous study, we have used hFLSCs expressing hypoxia inducible factor 1α (HIF-1α) to maintain self-renewal and pluripotency of hES cells [46]. Our present study also provided evidences that gene modified hFLSCs generated a stem cell niche in vitro that could support the growth and pluripotency of H9 hES cells. Our in vitro hES cells culture system using bFGF-hFLSCs as feeder cells provided an opportunity to elucidate the fundamental elements tightly controlling hES cell self-renewal and pluripotency.

In our present study, we aimed at determining whether the hES cells can be maintained pluripotent using bFGF-hFLSCs-CM rather than xenogenic MEF-CM. Similar to feeder-free culture in MEF-CM and mTeSR1, H9 hES cells can be equally maintained stable and pluripotent for over 15 passages (about 100 days). H9 hES cells maintained in bFGF-hFLSCs-CM without supplementing with bFGF retained typical morphology, expression of surface markers and transcription factors associated with pluripotency The conditioned medium of bFGF-hFLSCs could be used to develop a new hES cell feeder-free culture condition.

In conclusion, we isolated a new human feeder cell for maintenance of H9 hES cells. And then, we established transgenic cell lines — bFGF-hFLSCs that stably express bFGF, which could be used as feeder cells culturing H9 hES cells without supplementing with exogenous growth factors. More importantly of the bFGF-hFLSCs-CM could maintain H9 hES cell pluripotency in feeder-free culture conditions. bFGF-hFLSCs had great potential as feeders for maintaining hES cells more safely and economically.

## Supporting Information

Figure S1 The construction of lentiviral vector pBPLV.
(0.88 MB TIF)

Figure S2 H9 hES cells cultured on MEF. (A): Morphology of MEF. Bars  = 100 μm. (B): Morphology of H9 hES cells cultured on MEF. Bars  = 100 μm. (C): Immunophenotypic characterization of H9 hES cells cultured on MEF. Nuclei were stained with DAPI (blue). Bars: 100 μm. (D): *In vitro* differentiation of H9 hES cells cultured on MEF. RT-PCR analysis of various differentiation markers for the three germ layers and extraembryonic trophoblast.
(2.50 MB TIF)

Figure S3 Karyotype analysis of H9 hES cells expanded on hFLSCs feeder cells for 15 passages (about 100 days) represented normal 46, XX karyotype.
(1.12 MB TIF)

Figure S4 Transfected hFLSCs with low and high eGFP expression were sorted by fluorescence-activated cell sorting (FACS). The zonation of the selected transfected hFLSCs, P3 reprsented the hFLSCs with low eGFP expression, P4 represented hFLSCs with high eGFP expression.
(4.29 MB TIF)

Figure S5 Flow cytometry analysis of bFGF-hFLSCs for the presence of CD29, CD44, CD90, and CD105, and negative for CD11b, CD34, CD45 and CD144. The green line represented the staining with the isotype control, and the red line showed staining with specific antibodies.
(1.02 MB TIF)

Figure S6 Karyotype analysis of bFGF-hFLSCs for 20 passages (about 60 days) represented normal 46, XY karyotype.
(8.67 MB TIF)
