# Supplementary figures and images for: Human Fetal Liver Stromal Cells That Overexpress bFGF Support Growth and Maintenance of Human Embryonic Stem Cells
Source: PLoS One. 2010 Dec 30;5(12):e14457. doi: 10.1371/journal.pone.0014457 (PMC3012692; doi:10.1371/journal.pone.0014457)

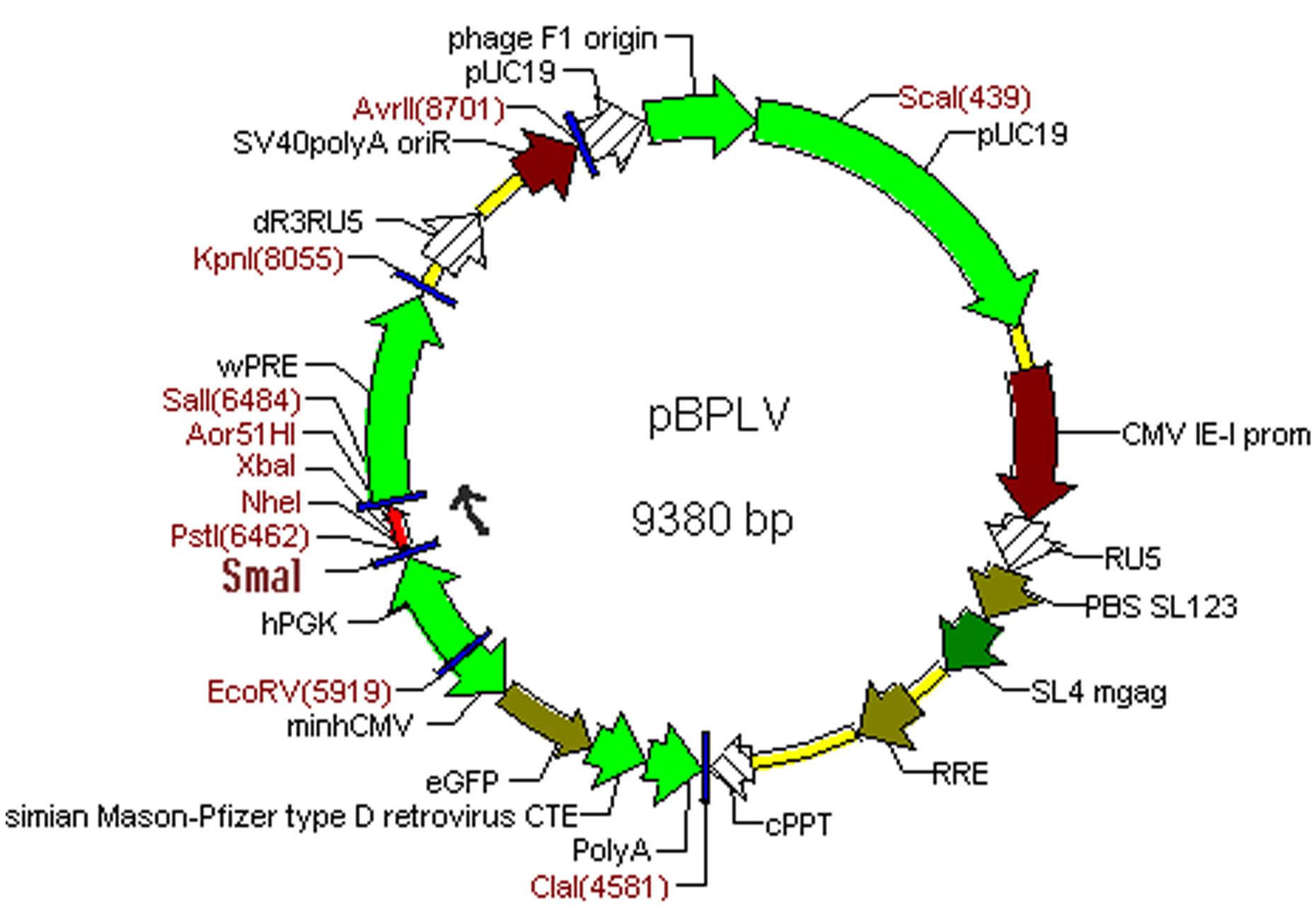

Supplement: Figure S1 — The construction of lentiviral vector pBPLV. (0.88 MB TIF) [file pone.0014457.s001.tif]

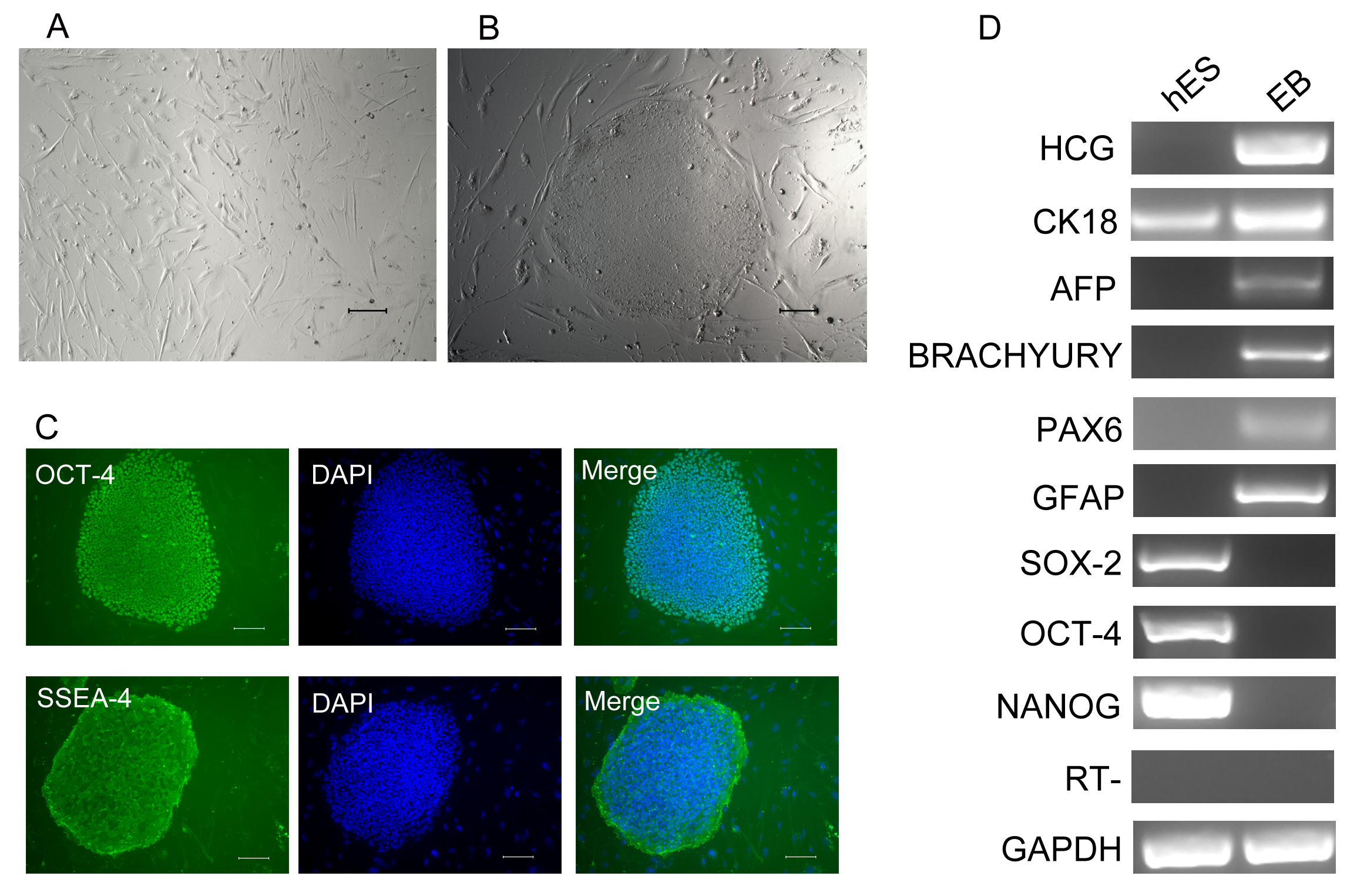

Supplement: Figure S2 — H9 hES cells cultured on MEF. (A): Morphology of MEF. Bars = 100 μm. (B): Morphology of H9 hES cells cultured on MEF. Bars = 100 μm. (C): Immunophenotypic characterization of H9 hES cells cultured on MEF. Nuclei were stained with DAPI (blue). Bars: 100 μm. (D): In vitro differentiation of H9 hES cells cultured on MEF. RT-PCR analysis of various differentiation markers for the three germ layers and extraembryonic trophoblast. (2.50 MB TIF) [file pone.0014457.s002.tif]

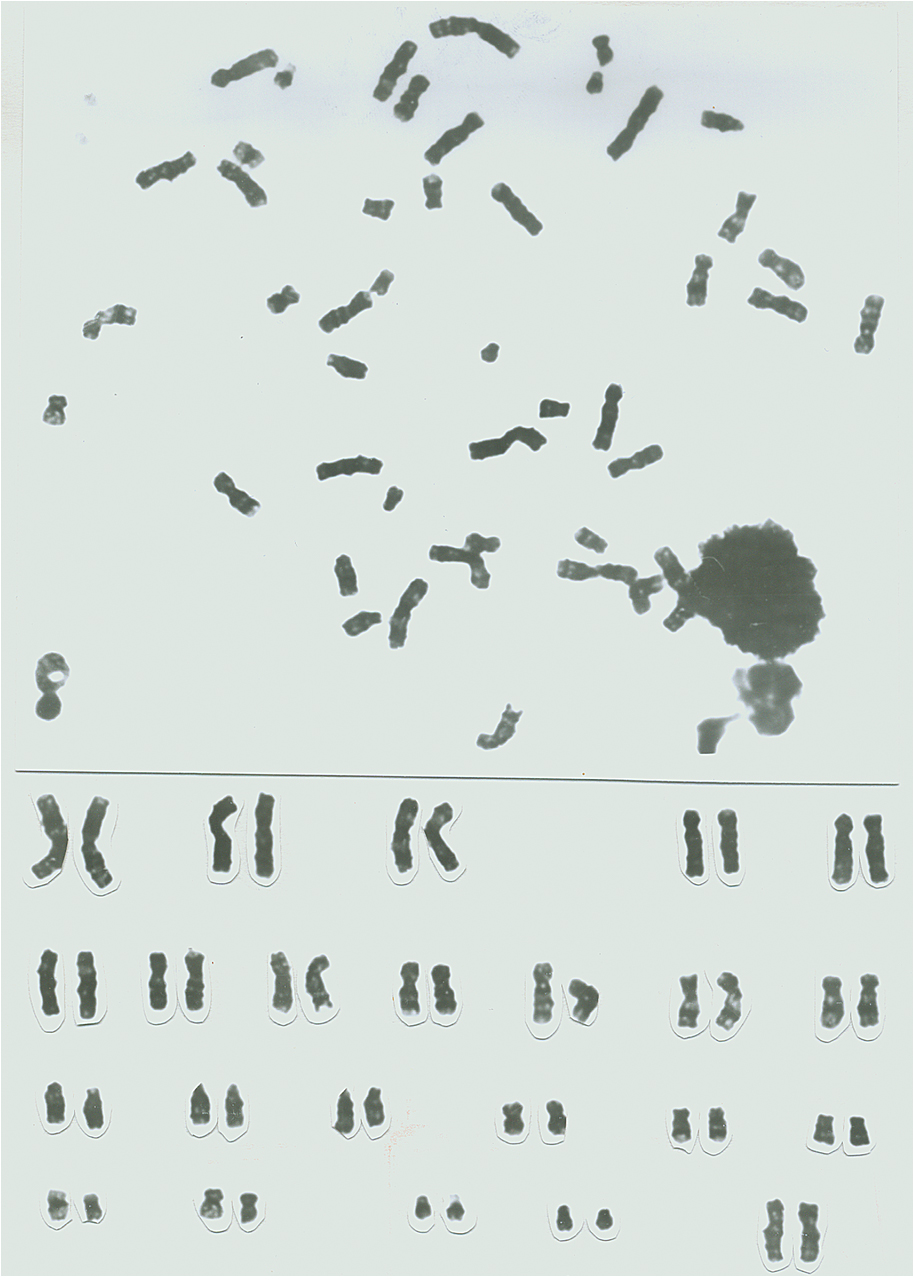

Supplement: Figure S3 — Karyotype analysis of H9 hES cells expanded on hFLSCs feeder cells for 15 passages (about 100 days) represented normal 46, XX karyotype. (1.12 MB TIF) [file pone.0014457.s003.tif]

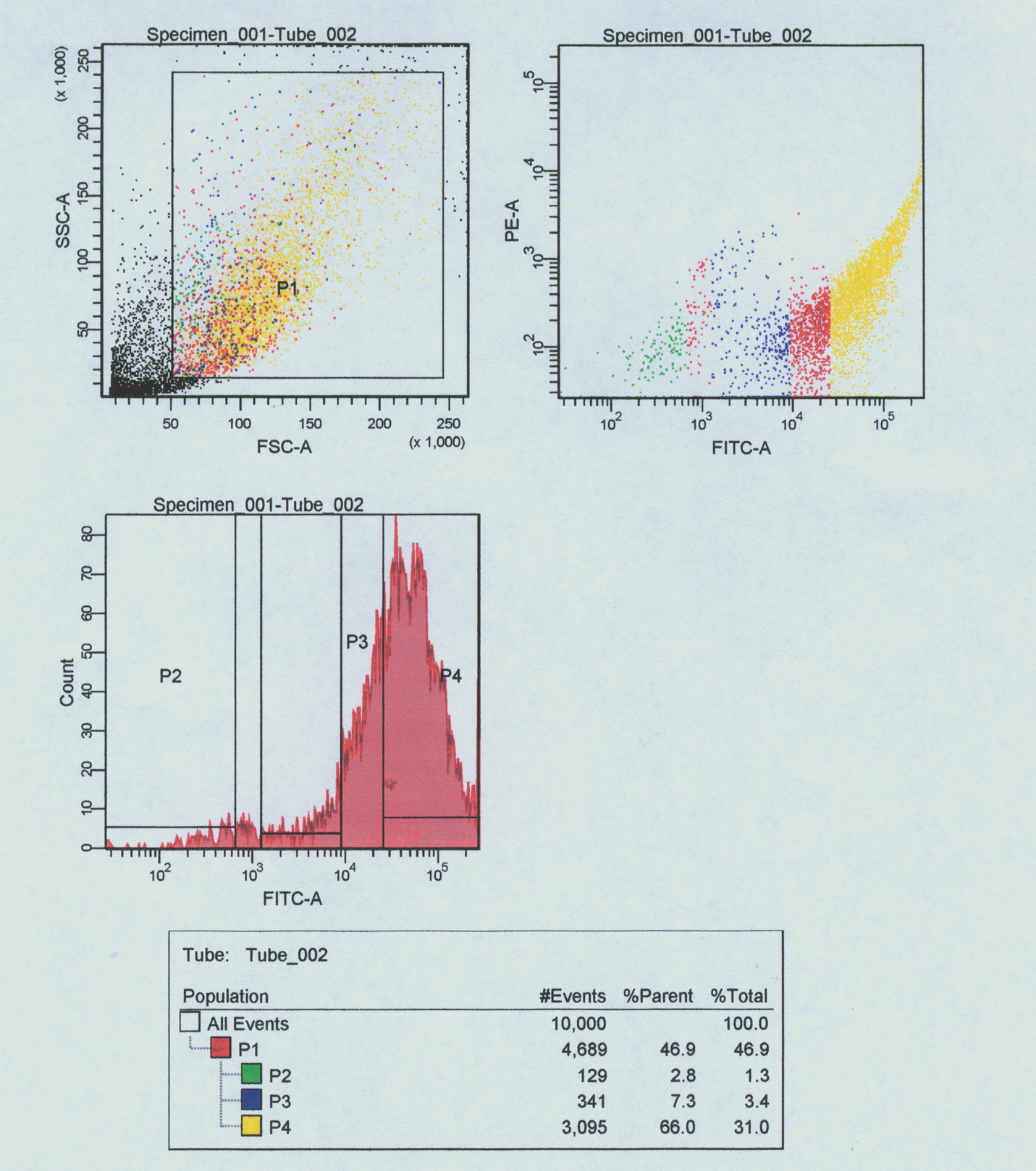

Supplement: Figure S4 — Transfected hFLSCs with low and high eGFP expression were sorted by fluorescence-activated cell sorting (FACS). The zonation of the selected transfected hFLSCs, P3 reprsented the hFLSCs with low eGFP expression, P4 represented hFLSCs with high eGFP expression. (4.29 MB TIF) [file pone.0014457.s004.tif]

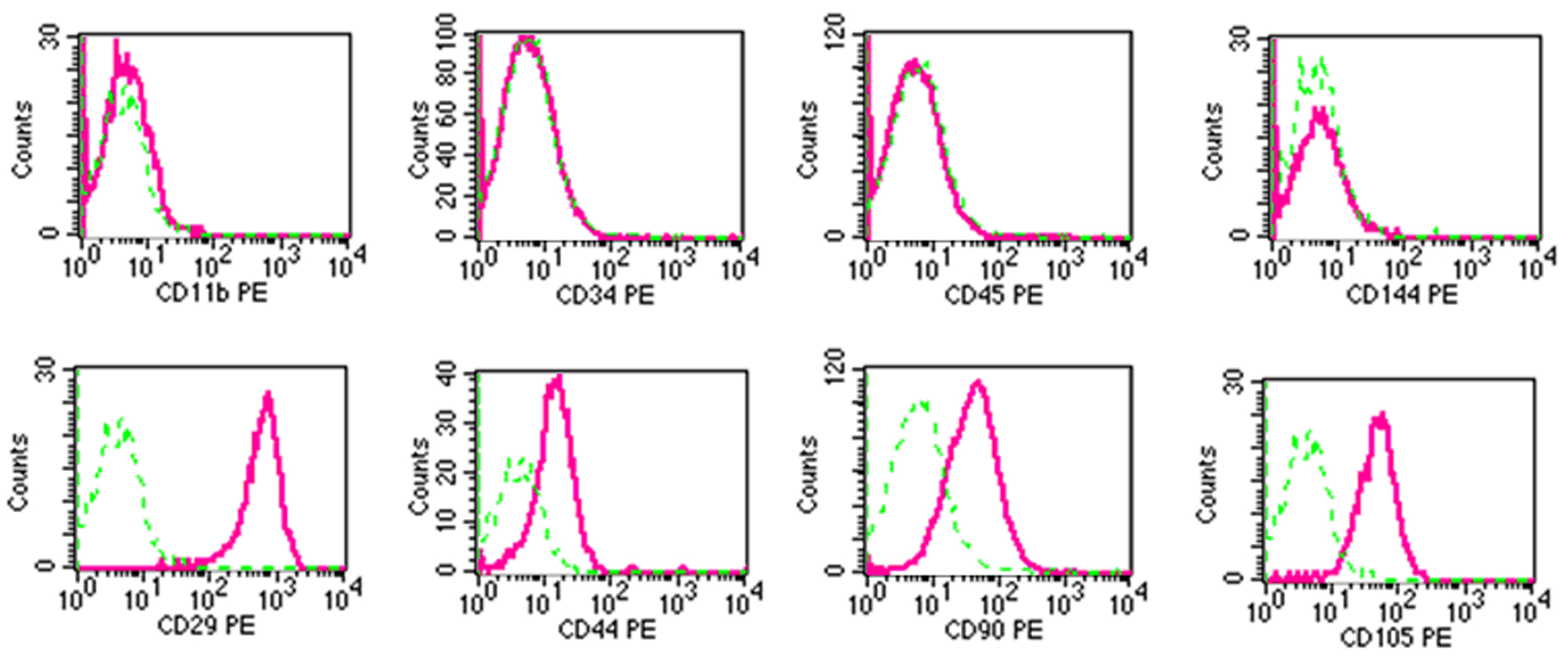

Supplement: Figure S5 — Flow cytometry analysis of bFGF-hFLSCs for the presence of CD29, CD44, CD90, and CD105, and negative for CD11b, CD34, CD45 and CD144. The green line represented the staining with the isotype control, and the red line showed staining with specific antibodies. (1.02 MB TIF) [file pone.0014457.s005.tif]

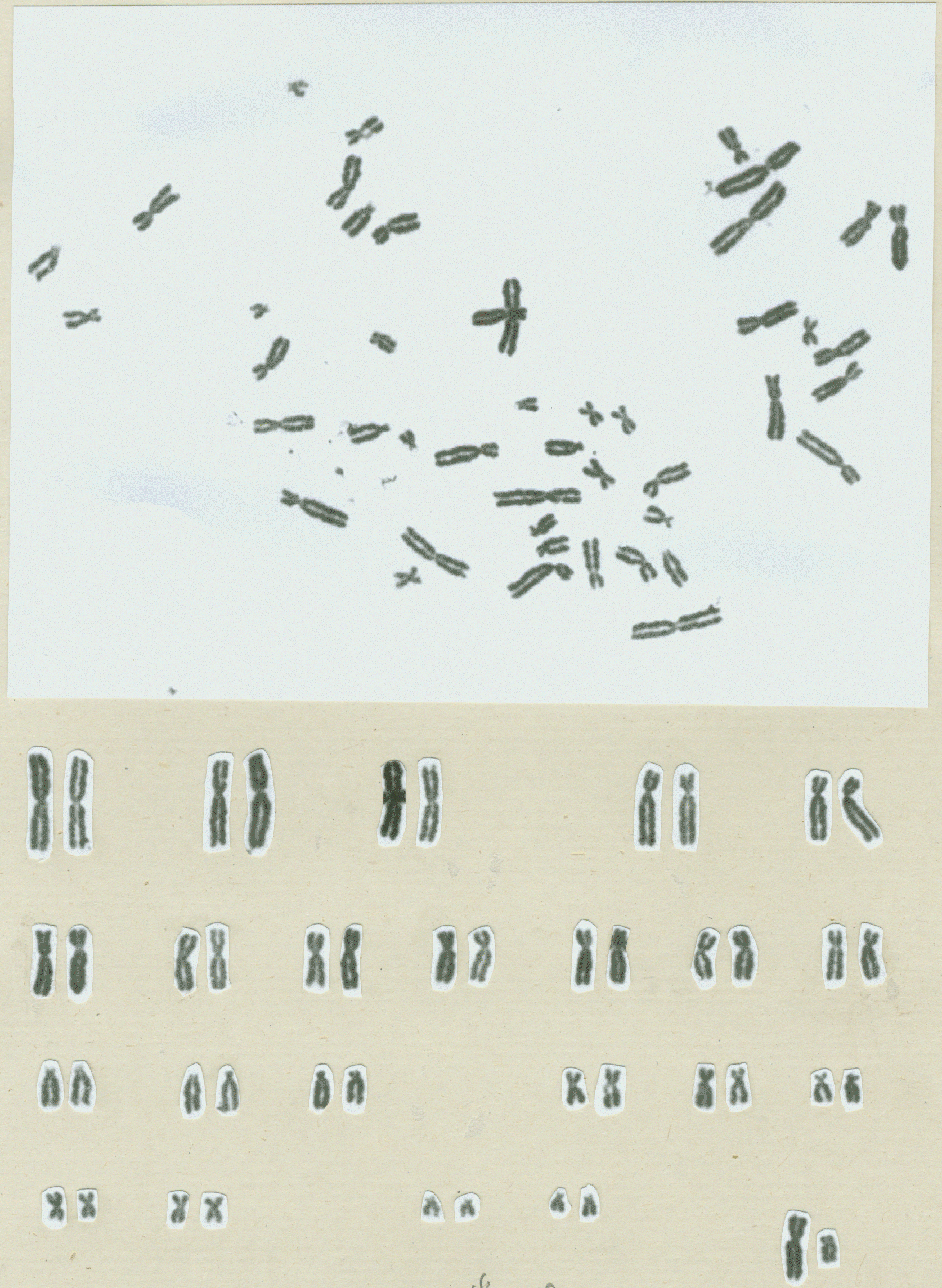

Supplement: Figure S6 — Karyotype analysis of bFGF-hFLSCs for 20 passages (about 60 days) represented normal 46, XY karyotype. (8.67 MB TIF) [file pone.0014457.s006.tif]
